# Presurgical computed tomography-guided localization of lung ground glass nodules: comparing hook-wire and indocyanine green

**DOI:** 10.1186/s12957-024-03331-7

**Published:** 2024-02-10

**Authors:** Rui Han, Long-Fei Wang, Fei Teng, Jia Lin, Yu-Tao Xian, Yun Lu, An-Le Wu

**Affiliations:** 1 Department of Interventional Radiology, The First Affiliated Hospital of Ningbo University, Ningbo, Zhejiang Province China; 2 Department of Thoracic Surgery, The First Affiliated Hospital of Ningbo University, Ningbo, Zhejiang Province China; 3 Department of Radiology, Xuzhou Central Hospital, Xuzhou, Jiangsu China

**Keywords:** Computed tomography, Indocyanine green localization, Hook-wire localization, Ground glass nodule

## Abstract

**Background:**

Presurgical computed tomography (CT)-guided localization is frequently employed to reduce the thoracotomy conversion rate, while increasing the rate of successful sublobar resection of ground glass nodules (GGNs) via video-assisted thoracoscopic surgery (VATS). In this study, we compared the clinical efficacies of presurgical CT-guided hook-wire and indocyanine green (IG)-based localization of GGNs.

**Methods:**

Between January 2018 and December 2021, we recruited 86 patients who underwent CT-guided hook-wire or IG-based GGN localization before VATS resection in our hospital, and compared the clinical efficiency and safety of both techniques.

**Results:**

A total of 38 patients with 39 GGNs were included in the hook-wire group, whereas 48 patients with 50 GGNs were included in the IG group. There were no significant disparities in the baseline data between the two groups of patients. According to our investigation, the technical success rates of CT-based hook-wire- and IG-based localization procedures were 97.4% and 100%, respectively (*P* = 1.000). Moreover, the significantly longer localization duration (15.3 ± 6.3 min vs. 11.2 ± 5.3 min, *P* = 0.002) and higher visual analog scale (4.5 ± 0.6 vs. 3.0 ± 0.5, *P* = 0.001) were observed in the hook-wire patients, than in the IG patients. Occurrence of pneumothorax was significantly higher in hook-wire patients (27.3% vs. 6.3%, *P* = 0.048). Lung hemorrhage seemed higher in hook-wire patients (28.9% vs. 12.5%, *P* = 0.057) but did not reach statistical significance. Lastly, the technical success rates of VATS sublobar resection were 97.4% and 100% in hook-wire and IG patients, respectively (*P* = 1.000).

**Conclusions:**

Both hook-wire- and IG-based localization methods can effectively identified GGNs before VATS resection. Furthermore, IG-based localization resulted in fewer complications, lower pain scores, and a shorter duration of localization.

**Supplementary Information:**

The online version contains supplementary material available at 10.1186/s12957-024-03331-7.

## Introduction

Computed tomography (CT)-guided lung cancer (LC) screening is a routine examination practiced globally [1]. According to a meta-analysis, low-dose CT screening improves the rate of stage I LC detection, while simultaneously reducing LC-related mortality rate [2]. Early stage LC typically presents as lung nodule on CT [3]. When radical intervention is needed to remove lung nodules, video-assisted thoracoscopic surgery (VATS) is typically recommended, and is associated with a shortened hospital stay and decreased morbidity compared to thoracotomy-based approaches [4]. In cases involving the management of unpalpable lung nodules, specifically subcentimeter nodules, deeper nodules, and ground glass nodules (GGNs), it has been observed that the rate of VATS conversion to thoracotomy is up to 63% [5, 6].

GGNs are nodules of slightly and homogeneously increased density with preserved bronchial and vascular margins found on high-resolution CT [7]. Currently, presurgical CT-guided localization is being increasingly used globally to decrease the rate of thoracotomy conversion, and simultaneously increase the rate of successful VATS sublobar (wedge or segmental) resection of lung nodules [6]. The most successful and frequently employed localization method is hook-wire localization, whose success rate is 94–98% [8]. However, the hook-wire method is associated with higher complication rate (up to 54%) [9]. In the past few years, medical practitioners have used liquid compounds, such as indocyanine green (IG), methylene blue, medical glue, and lipiodol for preoperative detection of lung nodules. This approach has demonstrated satisfactory safety and attainable outcomes [8–11]. However, there is noticeable scarcity of researchers comparing the localization of lung nodules, specifically GGNs, using hook-wire and IG techniques.

Therefore, this study aimed to evaluate the clinical effectiveness and safety of preoperative CT-guided hook-wire- and IG-based localization of GGNs.

## Methods

### Study design

This retrospective study received approval from the Institutional Review Board of First Affiliated Hospital of Ningbo University (No. 2023-077RS), and the participant consent requirement was waived. Between January 2018 and December 2021, we recruited 86 patients who undertook CT-guided hook-wire- or IG-based localization of GGNs before VATS resection in our hospital. Prior to January 2021, hook-wire was primarily used as localization material. The hospital transitioned to IG IG in January 2021, and ever since, this material has been utilized for GGN identification.

The following patients were eligible for analysis: (a) those with GGN; (b) maximal long-axis diameter ≤ 30 mm; and (c) age > 18 years old. The following patients were excluded from analysis: (a) maximal long-axis diameter < 5 mm; (b) diminished GGNs, as evidenced by CT-directed follow-up; and (c) the patients’ physical condition could not tolerate the VATS. The following were the indications for VATS resection of GGNs: those with (a) recent increase in size; (b) recent development or solid component enlargement; and (c) mixed GGN with solid components ≥ 6 mm.

### CT-guided hook-wire-based localization

We used a 16 Slice CT (Siemens, Berlin, Germany) to direct hook-wire localization according to the following parameters: 120 kV tube voltage, 100 mA tube current, 2 mm thickness, 0.6 s gantry rotation time, and 1.1 pitch.

**Fig. 1:**
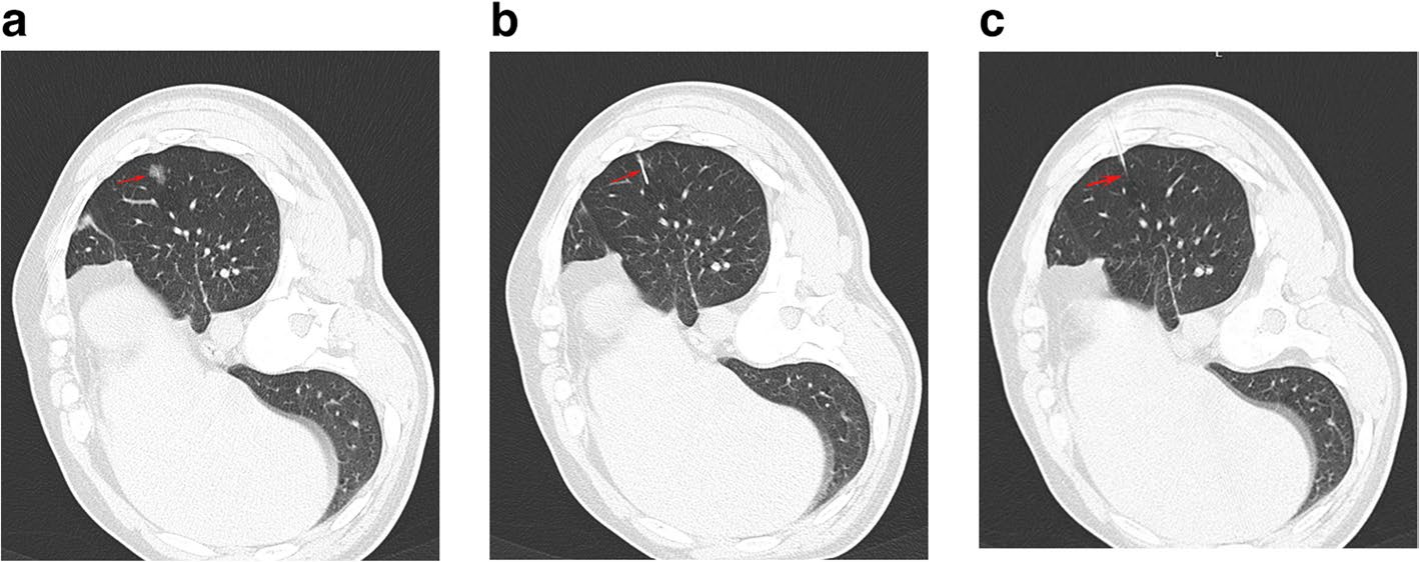
The CT-guided hook-wire GGN localization procedure. **a** CT images revealing GGN (arrow) at the left lower lobe. **b** Puncture needle (arrow) positioning near the GGN. **c** Hook-wire (arrow) positioning via the needle for GGN localization

Patients were positioned according to the GGN location (Fig. 1a). A needle path was chosen to reduce the distance between skin and GGN. Following the local anesthesia, a 21G needle (Argon Medical Device, Inc, TX, USA) was inserted into the lung parenchyma until the needle tip was within 10 mm of the GGN. The hook-wire was removed after CT-guided verification of the optimal needle placement (Fig. 1b). Postoperative CT imaging was utilized to validate the correct hook-wire identification and identify any potential procedural complications.

### CT-guided IG-based GGN localization

**Fig. 2:**
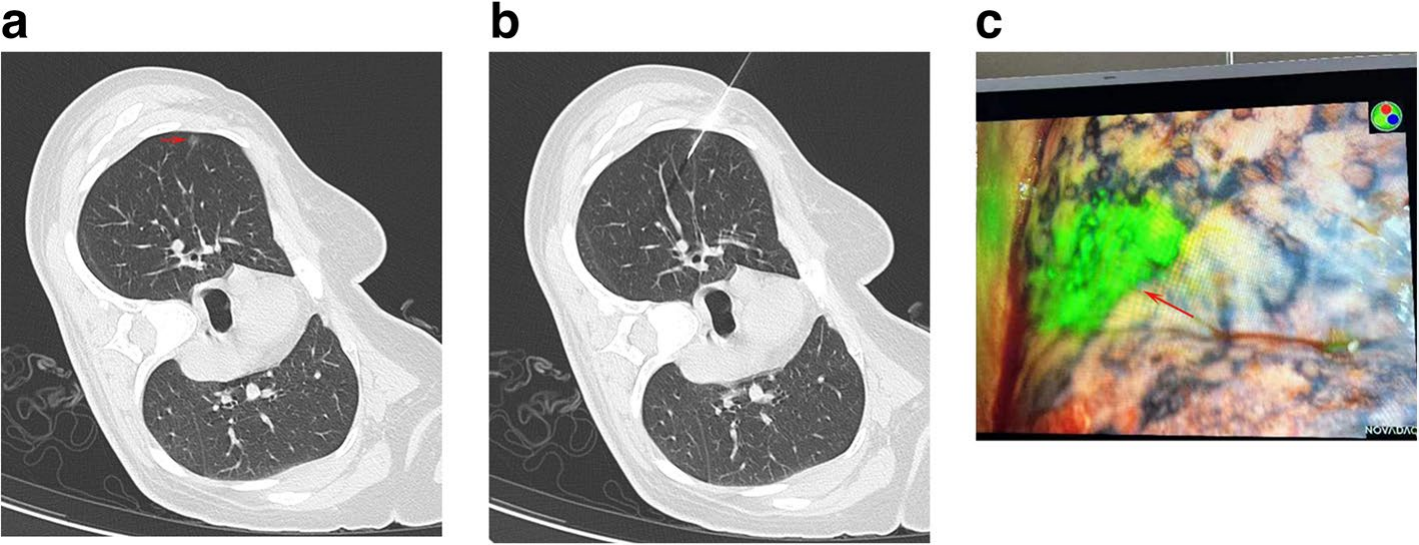
The CT-guided IG GGN localization procedure. **a** CT images revealing GGN (arrow) at the right upper lobe. **b** IG injection from the puncture needle (arrow) for GGN localization. **c** IG fluorescence (arrow) during the VATS resection

We employed the same CT parameters as the hook-wire localization protocol. Patients were positioned according to the GGN location (Fig. 2a). Once the needle tip (Fig. 2b) reached the point within 10 mm of GGN, the IG agent (2.5 mg/ml, 0.3 ml) was gently administered, and the needle was carefully removed such that the IG remained on the visceral pleura (Fig. 2c). Postoperative CT imaging was employed for detection of potential procedural complications.

### VATS resection

We performed VATS resection within 3 h of identification. Following the general anesthesia, we removed the GGN and localizer via wedge resection or segmentectomy according to the distance from the lesion to pleura. The segmentectomy was performed for a margin more than 2 cm from the edge of the lesion. In the cohort of patients who underwent hook-wire placement, the VATS resection procedure was guided by the viewing of the hook-wire. Among the patients in the IG group, the VATS resection procedure was guide by IG fluorescence visualization. The IG fluorescence was visualized using PINPOINT® endoscopic fluorescence imaging system (Novadaq, Mississauga, Canada). A surgical procedure, including wedge resection or segmentectomy, was conducted in cases where the margin distance from the edge of the lesion exceeded 2 cm. This procedure was carried out using a cutting suture technique. Subsequently, the excised lung tissue was forwarded for intra-operative frozen pathological assessment. A lobectomy and systemic lymph node dissection were conducted if the pathological diagnosis was invasive cancer. Lastly, we performed lymph node sampling for in situ or mini-invasive cancer.

### Assessment

Our primary endpoint was an identification-associated complication, and our secondary endpoint was a successful identification rate, sublobar resection rate, visual analog scale (VAS), intraoperative blood loss volume, and duration of post-surgical hospitalization. The technical success of hook-wire-based identification was determined by the visibility and stability of the hook-wire, while the technical success of IG identification was determined by the visibility of IG fluorescence on the visceral pleura, and its absence of diffusion from the injection site [12]. The sublobar resection technical success was described as the presence of the target GGN within the resected tissue. The VAS was measured immediately after the CT-guided identification procedure, and the patient answered the item “How do you rate your current respiratory status compared to the status before the intervention procedure?” [9] The VAS is presented as the 11-point (from 0 to 10) Box Scale (Supplement material).

### Statistical analyses

Data analyses employed SPSS 16.0 (SPSS, Inc., IL, USA), and data with normal distribution are expressed as means ± standard deviations, whereas, other data are provided as medians (Q1; Q3), and were respectively assessed using Student’s t- and Mann–Whitney U tests. Categorical data were assessed using chi-squared test or Fisher’s exact test. Lastly, multivariate logistic regression analysis was employed to identify the risk factors associated with pneumothorax and lung hemorrhage. *P* < 0.05 was deemed as significant.

## Results

### Patients’ characteristics

A total of 86 patients were included in this study. There were 38 patients (39 GGNs) who underwent hook-wire- and 48 patients (50 GGNs) who underwent IG-based localization (Table 1). The patients baseline information were comparable between 2 groups.

**Table 1:** Baseline characteristics between 2 groups

	Hook-wire group	IG group	*P*
Patients number	38	48	-
Age (y)	46.5 ± 11.1	47.8 ± 12.7	0.621
Gender			0.933
Male	9	11	
Female	29	37	
Nodule number			1.000
Single	37	46	
Multiple	1	2	
Diameter (mm)	7.1 ± 2.0	6.7 ± 1.9	0.401
Nodule-pleura distance (mm)	7.5 (Q1: 2.9; Q3: 13.0)	7.8 (Q1: 4.0; Q3: 15.0)	0.508
Nature of the nodules			0.430
Pure GGN	32	44	
Mixed GGN	7	6	
Location of the nodules			0.612
Right upper	9	12	
Right middle	0	3	
Right lower	10	10	
Left upper	10	13	
Left lower	10	12	

*GGN* Ground glass nodule, *IG* Indocyanine green

### CT-guided GGN localization results

The technical success rates of CT-guided hook-wire- and IG-based localization were 97.4% and 100%, respectively (*P* = 1.000, Table 2). In one GGN, we experienced technical failure as the hook-wire dislodged. The significantly longer localization duration (15.3 ± 6.3 min vs. 11.2 ± 5.3 min, *P* = 0.002) and higher VAS (4.5 ± 0.6 vs. 3.0 ± 0.5, *P* = 0.001) were observed among the patients in hook-wire group, relative to the patients in IG group.

**Table 2:** Comparison of localization-related data

	Hook-wire	Indocyanine green	*P*
Technical success rate	97.4% (38/39)	100% (50/50)	1.000
Dislodgement	1	Not applicable	-
Duration of localization (min)	15.3 ± 6.3	11.2 ± 5.3	0.002
VAS	4.5 ± 0.6	3.0 ± 0.5	0.001
Complications			
Pneumothorax	23.7% (9/38)	6.3% (3/48)	0.048
Lung hemorrhage	28.9% (11/38)	12.5% (6/48)	0.057

*VAS* Visual analog scale

### Localization related complications

Pneumothorax occurred in 9 (23.7%) and 3 (6.3%) hook-wire and IG patients, respectively (*P* = 0.048). Table 3 presents the predictors of pneumothorax. The results of our univariate logistic regression analysis indicate a significant correlation between non-upper lobe (*P* = 0.02), longer identification duration (*P* = 0.013), and hook-wire identification (*P* = 0.03) with pneumothorax. When these factors were combined into the multivariate logistic analysis, we revealed that the stand-alone pneumothorax risk factors were prolonged identification duration (*P* = 0.041) and non-upper lobe (*P* = 0.015).

**Table 3:** Predictors of pneumothorax

	Univariate analysis			Multivariate analysis		
	Hazard ratio	95% CI	*P* value	Hazard ratio	95% CI	*P* value
Age	0.967	0.917–1.020	0.217			
Gender						
Male	1					
Female	0.895	0.217–3.681	0.878			
Diameter	1.010	0.738–1.381	0.952			
Nodule-pleura distance	0.965	0.889–1.047	0.39			
Nature of the nodules						
Pure GGN	1					
Mixed GGN	0.470	0.055–3.986	0.499			
Lung sides						
Right	1					
Left	0.425	0.118–1.535	0.192			
Lung lobes						
Non-upper	1			1		
Upper	0.152	0.031 – 0.744	0.02	0.116	0.020–0.656	0.015
Duration of localization	1.128	1.026 – 1.241	0.013	1.130	1.005–1.270	0.041
Localization material						
Hook-wire	1			1		
IG	0.215	0.054 – 0.860	0.03	0.324	0.070–1.502	0.150

*GGN* Ground glass nodule, *IG* Indocyanine green

Lung hemorrhage occurred in 11 (28.9%) and 6 (12.5%) hook-wire and IG patients, respectively (*P* = 0.057). The logistic analysis revealed that no risk factors were independently connected to lung hemorrhage (Table 4).

**Table 4:** Predictors of lung hemorrhage

	Univariate analysis			Multivariate analysis		
	Hazard ratio	95% CI	*P* value	Hazard ratio	95% CI	*P* value
Age	1.009	0.965–1.055	0.694			
Gender						
Male	1					
Female	0.981	0.280–3.433	0.976			
Diameter	1.003	0.764–1.318	0.981			
Nodule-pleura distance	1.011	0.955–1.071	0.698			
Nature of the nodules						
Pure GGN	1			1		
Mixed GGN	3.177	0.886–11.396	0.076	2.986	0.805–11.080	0.102
Lung sides						
Right	1					
Left	1.471	0.502–4.309	0.482			
Lung lobes						
Non-upper	1					
Upper	0.605	0.207 – 1.775	0.360			
Duration of localization	1.015	0.931 – 1.106	0.733			
Localization material						
Hook-wire	1			1		
IG	0.351	0.116 – 1.060	0.063	0.366	0.119 – 1.126	0.080

*GGN* Ground glass nodule, *IG* Indocyanine green

### VATS results

The VATS sublobar resection was successfully performed for all GGNs which were successfully localized (Table 5). A direct VATS lobectomy was conducted for technical failure of localization of a GGN with the hook-wire due to hook-wire displacement. We observed no obvious differences in the VATS duration (76.2 ± 37.6 min vs. 84.8 ± 43.2 min, *P* = 0.336), surgical categories, blood loss volume (15 ml vs. 10 ml, *P* = 0.114), final diagnoses, or duration of postsurgical hospitalization (8.0 ± 3.7 d vs. 7.8 ± 1.9 d, *P* = 0.654) between the hook-wire and IG patients (Table 5).

**Table 5:** Comparison of VATS-related data

	Hook-wire group	IG group	*P*
Technical success of sublobar resection	97.4% (38/39)	100% (50/50)	1.000
Duration of VATS (min)	76.2 ± 37.6	84.8 ± 43.2	0.336
Surgical types			0.291
Wedge resection	25	25	
Segmentectomy	10	21	
Wedge resection + lobectomy	3	4	
Direct lobectomy	1	0	
Blood loss (ml)	15 (Q1:5; Q3: 20)	10 (Q1: 10; Q3: 17.5)	0.114
Final diagnoses			0.711
Invasive adenocarcinoma	3	4	
Mini-invasive adenocarcinoma	23	31	
Adenocarcinoma in situ	10	10	
Precancerous lesion	1	4	
Benign	2	1	
Post-operative hospital stay (d)	8.0 ± 3.7	7.8 ± 1.9	0.654

*IG* Indocyanine green, *VATS* Video‑assisted thoracoscopic surgery

## Discussion

Detecting GGNs during VATS resection is challenging due to their frequently nonpalpable nature [13–15]. Therefore, accurately detecting GGNs is an essential stage in VATS resection. Preoperative GGN identification not only allows for precise resection, but also helps avoid unnecessary extensive resections in patients with GGNs [15]. Besides hook-wire- and liquid-based materials, micro-coil and radio-label are employed for presurgical GNN localization [7, 16–18]. Nevertheless, the microcoil localization technique is comparatively more complex than the liquid material and hook-wire techniques due to the requirement to maintain the microcoil's end tail above the visceral pleura [16]. Furthermore, the utilization of radio-label-based localization requires the use of intraoperative fluoroscopic guidance, which has the potential to result in radiation-induced damage [17, 18].

In this study, we determined the localization success rates of hook-wire- and IG-based methods were both considerably enhanced, with rates of 97.4% and 100%, respectively (*P* = 1.000). The findings of our study were in consistent with those of previous research comparing hook-wire and IG localization methods [9], as well as those comparing hook-wire and methylene blue localization in the context of lung nodules [19]. The observed higher success rates of hook-wire and IG localization can be attributed to the detectability of these localization materials.

The dye localization materials mainly include IG and methylene blue [12, 19]. These 2 materials are commonly used for detecting the sentinel lymph nodes in breast cancer [20]. IG can exhibit higher detection rate and better accuracy than methylene blue in detecting sentinel lymph nodes because IG has the better affinity to the lymph nodes [20]. However, when using the IG and methylene blue for localization of lung nodules, there is no significant difference in technical aspects [21].

The technical failure of hook-wire- and IG-based localization is primarily caused by hook-wire dislodgement and IG diffusion. The hook-wire is commonly positioned within the lung parenchyma at an angle that allows it to protrude through the chest wall. Unfortunately, this results in dislodgement or migration during respiratory movement [12]. The key factor of IG-based localization is the injected IG volume. Several reports suggested that 0.3 ml IG is sufficient for lung nodule localization [12]. Upon injection of excess IG volume, the IG material can overflow. Alternately, < 0.3 ml may not be sufficient for proper localization [12].

The analysis strongly focuses on the crucial endpoints associated with localization-related complications. In this study, the IG-based localization method had a lower incidence of pneumothorax and lower VAS scores than the hook-wire-based localization method. There is a substantial relationship between hook-wire dislodgement and increased incidences of pneumothorax, lung hemorrhage, and chest discomfort [12]. However, the pneumothorax risk factors in this study were prolonged localization duration and non-upper lobe, and not hook-wire usage. This result may be due to our small sample population. Regardless, we demonstrated that hook-wire-based localization took significantly longer duration than IG-based localization. This indicates that hook-wire usage may trigger pneumothorax development. Non-upper lobe lesion is another risk factor for pneumothorax, and this finding may be because, relative to the upper lung fields, the lower lung field participates in more respiratory motion [22].

The VATS outcomes are generally independent of various localization materials [10, 12, 15]. In this study, we demonstrated that the rate of VATS sublobar resection, blood loss volume, and postsurgical hospitalization duration were comparable in both patient cohorts. Additionally, a significantly larger proportion of GGN patients (91.9%, 79/86) presented with pathological diagnoses below the level of invasive adenocarcinoma, preventing the need for lobectomy. Hence, the median blood loss volume was only 15 ml and 10 ml among the hook-wire and IG patients, respectively.

Although we have found some superiorities of IG when compared to hook-wire in this study, IG also has its own limitations [13]. First of all, the IG dye is prone to diffusion, resulting in failure of localization [13]. Secondly, IG localization is only marked on the surface of the lung, so we should judge the depth of the GGN according to the preoperative CT results. In contrast, hook-wire is partially inserted into the lung parenchyma, and thus we can directly judge the depth of GGN according to palpation of the hook-wire during the VATS.

This work has certain limitations. First, it is a retrospective study. Consequently, while using comparable baseline information can help reduce the risk of selection bias, it is important to note that the feasibility and safety of hook-wire and IG procedures may vary in different contexts. Therefore, it is necessary to conduct further well-designed prospective randomized trials. Second, our sample population was relatively small. Therefore, the risk factor for lung hemorrhage was not determined. Additionally, it is important to note that the patients included in this study were obtained exclusively from a single center. Consequently, it is recommended that further research be conducted with multiple facilities to ensure the generalization and reliability of these results.

## Conclusion

In conclusion, our findings demonstrate that both hook-wire- and IG-based localization methods can effectively identify GGNs before VATS resection. Furthermore, IG-based localization resulted in fewer complications, lower pain scores, and a shorter duration of localization.

### Supplementary Information

**Additional file 1.**

## Data Availability

The data that support the findings of this study are available from the corresponding author upon reasonable request.

## Abbreviations

CT: Computed tomography
GGN: Ground glass nodule
IG: Indocyanine green
VATS: Video-assisted thoracoscopic surgery
