# Supplementary material for: Presurgical computed tomography-guided localization of lung ground glass nodules: comparing hook-wire and indocyanine green
Source: World J Surg Oncol. 2024 Feb 10;22:51. doi: 10.1186/s12957-024-03331-7 (PMC10858508; doi:10.1186/s12957-024-03331-7)
Supplement: Supplementary file 1 — Additional file 1. [file 12957_2024_3331_MOESM1_ESM.doc]

No pain+----+----+----+----+----+----+----+----+----+----+Pain as bad as it could be

0 10

0 score: No pain, no feel of pain;

1-3 scores: Mild pain, no influence of life or work;

4-6 scores: Moderate pain, influence of work, while no influence of life;

7-10 scores: Severe pain, extremely pain, influence of work and life.
